# Modelling the impact of a tax on sweetened beverages in the Philippines: an extended cost–effectiveness analysis

**DOI:** 10.2471/BLT.18.219980

**Published:** 2018-12-05

**Authors:** Akshar Saxena, Adam D Koon, Leizel Lagrada-Rombaua, Imelda Angeles-Agdeppa, Benjamin Johns, Mario Capanzana

**Affiliations:** a Department of Global Health and Population, Harvard T.H. Chan School of Public Health, Boston, United States of America (USA).; b International Development Division, Abt Associates Inc., 6130 Executive Blvd, Rockville, MD 20852, USA.; c Independent Consultant, Block 14 Lot 4 Lapulapu Street, New Capitol Estates 1, Batasan Hills, Quezon City, Philippines 1126.; d Food and Nutrition Research Institute, Department of Science and Technology, Manila, Philippines.

## Abstract

**Objective:**

To assess the potential impact of a new tax on sweetened beverages on premature deaths associated with noncommunicable diseases in the Philippines.

**Methods:**

In January 2018, the Philippines began imposing a tax of 6 Philippine pesos per litre (around 13%) on sweetened beverages to curb the obesity burden. Using national data sources, we conducted an extended cost–effectiveness analysis to estimate the effect of the tax on the numbers of premature deaths averted attributed to type 2 diabetes mellitus, ischaemic heart disease and stroke, across income quintiles over the period 2018–2037. We also estimated the financial benefits of the tax from reductions in out-of-pocket payments, direct medical costs averted and government health-care cost savings.

**Findings:**

The tax could avert an estimated 5913 deaths related to diabetes, 10 339 deaths from ischaemic heart disease and 7950 deaths from stroke over 20 years. The largest number of deaths averted could be among the fourth and fifth (highest) income quintiles. The tax could generate total health-care savings of 31.6 billion Philippine pesos (627 million United States dollars, US$) over 20 years, and raise 41.0 billion Philippine pesos (US$ 813 million) in revenue per annum. The poorest quintile could bear the smallest tax burden increase (14% of the additional tax; 5.6 billion Philippine pesos) and have the lowest savings in out-of-pocket payments due to relatively large health-care subsidies. Finally, we estimated that 13 890 cases of catastrophic expenditure could be averted.

**Conclusion:**

The new sweetened beverage tax may help to reduce obesity-related premature deaths and improve financial well-being in the Philippines.

## Résumé

**Objectif:**

Évaluer le potentiel impact d'une nouvelle taxe sur les boissons sucrées sur les décès prématurés associés à des maladies non transmissibles aux Philippines.

**Méthodes:**

En janvier 2018, les Philippines ont commencé à imposer une taxe de 6 pesos philippins par litre (environ 13%) sur les boissons sucrées afin de réduire le fardeau de l'obésité. À partir de sources de données nationales, nous avons réalisé une analyse coût-efficacité approfondie pour estimer l'effet de cette taxe sur le nombre de décès prématurés évités imputables au diabète sucré de type 2, à une maladie cardiaque ischémique ou à un accident vasculaire cérébral, pour tous les quintiles de revenu, sur la période 2018-2037. Nous avons également estimé les avantages financiers de la taxe découlant de la diminution des débours, des coûts médicaux directs évités et des économies de coûts des soins de santé réalisées par le gouvernement.

**Résultats:**

Nous avons estimé que sur 20 ans, la taxe permettrait d'éviter 5913 décès liés au diabète, 10 339 décès dus à une maladie cardiaque ischémique et 7950 décès attribuables à un accident vasculaire cérébral. Le plus grand nombre de décès évités pourrait se situer dans les quatrième et cinquième quintiles de revenu (les plus hauts). La taxe pourrait générer des économies totales en soins de santé de 31,6 milliards de pesos philippins (627 millions de dollars des États-Unis) sur 20 ans, et rapporter des recettes de 41,0 milliards de pesos philippins (813 millions de dollars des États-Unis) par an. Le quintile le plus pauvre pourrait supporter la plus faible augmentation de la charge fiscale (14% de la taxe additionnelle; 5,6 milliards de pesos philippins) et afficher la plus faible diminution de débours en raison de subventions pour les soins de santé relativement élevées. Enfin, nous avons estimé que 13 890 cas de dépenses ruineuses pourraient être évités.

**Conclusion:**

La nouvelle taxe sur les boissons sucrées pourrait contribuer à diminuer le nombre de décès prématurés liés à l'obésité et améliorer le bien-être financier aux Philippines.****

## Resumen

**Objetivo:**

Evaluar el impacto potencial de un nuevo impuesto a las bebidas endulzadas en las muertes prematuras asociadas con las enfermedades no contagiosas en Filipinas.

**Métodos:**

En enero de 2018, Filipinas comenzó a imponer un impuesto de 6 pesos filipinos por litro (alrededor de un 13 %) a las bebidas endulzadas para reducir la carga de la obesidad. A partir de fuentes de datos nacionales, se realizó un análisis de ampliado de coste y efectividad para estimar el efecto del impuesto en el número de muertes prematuras evitadas atribuidas a la diabetes mellitus tipo 2, la cardiopatía isquémica y el accidente cerebrovascular, en todos los quintiles de ingresos durante el periodo 2018-2037. También se estimaron los beneficios financieros del impuesto a partir de las reducciones en los pagos directos, los costes médicos directos evitados y los ahorros en los costes de la atención sanitaria para el gobierno.

**Resultados:**

El impuesto podría evitar unas 5913 muertes relacionadas con la diabetes, 10339 muertes por cardiopatía isquémica y 7950 muertes por accidente cerebrovascular en 20 años. El mayor número de muertes evitadas podría encontrarse entre los quintiles cuarto y quinto (más altos) de ingresos. El impuesto podría generar un ahorro total en la atención sanitaria de 31 600 millones de pesos filipinos (627 millones de USD) en 20 años y recaudar hasta 41 000 millones de pesos filipinos (813 millones de USD) en ingresos anuales. El quintil más pobre podría soportar el menor aumento de la carga tributaria (14 % del impuesto adicional; 5600 millones de pesos filipinos) y ser quien menos ahorrase en los pagos directos debido a los subsidios relativamente grandes para la atención sanitaria. Por último, se estima que podrían evitarse unos 13 890 casos de gastos catastróficos.

**Conclusión:**

El nuevo impuesto a las bebidas endulzadas podría ayudar a reducir las muertes prematuras relacionadas con la obesidad y mejorar el bienestar financiero en Filipinas.

## ملخص

**الغرض:**

تقييم الأثر المحتمل لفرض ضريبة جديدة على المشروبات المحلاة على الوفيات المبكرة المرتبطة بالأمراض غير المعدية في الفلبين.

**الطريقة:**

في يناير/كانون ثاني 2018، بدأت الفلبين فرض ضرائب بقيمة 6 بيزو فلبيني لكل لتر (حوالي 13٪) على المشروبات المحلاة للحد من عبء السمنة. باستخدام مصادر البيانات الوطنية، قمنا بإجراء تحليل موسع لمدى فعالية التكلفة وذلك لتقدير أثر الضرائب على أعداد الوفيات المبكرة التي تم تفاديها والتي تعزى إلى النوع الثاني من داء السكري ومرض القلب الإقفاري والسكتة الدماغية، عبر خماسية الدخل خلال الفترة 2018 إلى 2037. كما قدرنا الفوائد المالية للضرائب والناتجة عن التخفيضات في مدفوعات الجيب، والتكاليف الطبية المباشرة التي تم تفاديها، ومقدار التوفير في تكاليف الرعاية الصحية الحكومية.

**النتائج:**

أدت الضريبة إلى تجنب ما يقدر بنحو 5913 حالة وفاة مرتبطة بداء السكري، و10339 حالة وفاة بسبب مرض القلب الإقفاري، و7950 حالة وفاة بسبب السكتة الدماغية على مدى 20 عاما. يمكن أن يكون أكبر عدد من الوفيات التي تم تفاديها بين الخُمسيْن الرابع والخامس (الأعلى) من الدخل. ويمكن أن تؤدي الضرائب إلى إجمالي من التوفير في الرعاية الصحية قدره 31.6 مليار بيزو (627 مليون دولار أمريكي) على مدى 20 عاماً، كما تؤدي لزيادة في العائد بقيمة 41.0 مليار بيزو (813 مليون دولار أمريكي) سنوياً. يمكن لأفقر خُمس أن يتحمل أصغر زيادة في عبء الضرائب (14٪ من الضرائب الإضافية؛ 5.6 مليار بيزو) ولديها أقل مستوى من مدخرات في مدفوعات الجيب بسبب الدعم الضخم نسبياً للرعاية الصحية. وفي النهاية، قدرنا أنه يمكن تفادي 13890 حالة من النفقات الكارثية.

**الاستنتاج:**

قد تساعد الضرائب الجديدة على المشروبات المحلاة على تقليل الوفيات المبكرة المرتبطة بالسمنة، وتحسين الرفاهية المالية في الفلبين. ****

## 摘要

**目的:**

旨在评估菲律宾对甜味饮料征收新税与非传染性疾病过早死亡间的潜在影响。

**方法:**

2018 年 1 月，菲律宾开始对甜味饮料征收每升 6 菲律宾比索（约 13％）的消费税，以遏制肥胖负担。我们采用扩展成本效益分析法来分析国家数据来源，研究对 2018 至 2037 年间收入五等分位的人群征税，并估计由于征税而避免因 II 型糖尿病、缺血性心脏病和中风过早死亡的人数。我们还通过减少自付费用、避免直接医疗费用和节省政府医疗费用来估算税收的经济效益。

**结果:**

20 年内，征税可避免约 5913 人死于糖尿病，10，339 人死于缺血性心脏病，7950 人死于中风。因征税政策而免于死亡的人数最多的人群可能位于第四和第五（最高）收入五分位。20 年内，征税可节约总计 316 亿比索（6.27 亿美元，US$）的医疗保健额，并且每年的税收收入可提高 410 亿比索（8.13 亿美元）。最贫困的五分之一人群可以承担最低的税负增加（额外税收的 14％；56 亿比索），并且由于相对较高的医疗补贴，自付费用最低。最后，我们估计可避免 13，890 起灾难性卫生支出案例。

**结论:**

对甜味饮料征收新税可能有助于减少与肥胖相关的过早死亡，从而改善菲律宾的健康状况。

## Резюме

**Цель:**

Оценить потенциальное влияние нового акциза на подслащенные напитки на показатели преждевременной смертности на Филиппинах, связанной с неинфекционными заболеваниями.

**Методы:**

В январе 2018 года Филиппины ввели акциз в размере 6 филиппинских песо за литр (около 13%) подслащенных напитков с целью уменьшения числа людей, страдающих ожирением. Используя национальные источники данных, авторы провели расширенный анализ экономической эффективности по квинтилям дохода за период 2018–2037 гг., чтобы оценить влияние акциза на преждевременную смертность, связанную с сахарным диабетом 2-го типа, ишемической болезнью сердца и инсультом. Авторы также оценили экономическую выгоду от введения акциза за счет сокращения собственных расходов пациентов на медицинское обслуживание, предотвращения прямых медицинских расходов и сокращения государственных расходов на здравоохранение.

**Результаты:**

Введение акциза могло бы предотвратить предположительно 5913 смертных случаев, связанных с диабетом, 10 339 смертных случаев от ишемической болезни сердца и 7950 смертных случаев от инсульта в течение 20 лет. Предотвращение наибольшего числа смертных случаев может произойти среди четвертого и пятого (самого высокого) квинтилей дохода. В целом введение акциза поможет сэкономить 31,6 млрд филиппинских песо (627 млн долл. США) в течение 20 лет за счет сокращения расходов на здравоохранение и увеличить ежегодный доход страны на 41,0 млрд филиппинских песо (813 млн долл. США). Для самого бедного квинтиля увеличение налоговой нагрузки будет минимальным (14% от дополнительного акциза, 5,6 млрд филиппинских песо), а экономия собственных средств пациентов достигнет самого низкого показателя из-за относительно больших субсидий на здравоохранение. Кроме того, авторы считают, что можно предотвратить 13 890 случаев запредельных расходов на здравоохранение.

**Вывод:**

Новый акциз на подслащенные напитки поможет снизить преждевременную смертность, связанную с ожирением, и улучшить финансовое благополучие на Филиппинах.****

## Introduction

Sugar-sweetened beverages are a driver of obesity,^1^^–^^4^ and increasingly contribute to the burden of noncommunicable disease in low- and middle-income countries.^5^ This is particularly true in the Philippines, where 31.1% (17.5 million) of the 56.3 million adults in 2013 were overweight and the percentage of overweight youth has nearly doubled, from 4.9% (0.9 million of 18.5 million) to 8.3% (1.7 million of 20.3 million), in 10 years.^6^ This has left health officials looking for strategies to mitigate the burden of obesity.

On 19 December 2017, the Tax Reform for Acceleration and Inclusion Act was signed into law and was implemented in January 2018. This included a 6 Philippine pesos per litre excise tax on sweetened beverages made with caloric or non-caloric sweeteners and a 12 Philippine pesos per litre tax on beverages made with high-fructose corn syrup (equivalent to 0.12 United States dollars, US$, and US$ 0.24 in January 2018, respectively). This two-tiered levy represented retail price increases of approximately 13% from 45 to 51 Philippine pesos per litre of regular cola and 26% from 45 to 57 Philippine pesos per litre of cola made with high-fructose corn syrup, respectively. Milk, 100% natural fruit juice and 3-in-1 instant coffee were excluded.

The Philippines is one of 27 countries that has introduced a sweetened beverage tax, joining others such as Chile, France, Mexico, Spain and six municipalities in the United States of America.^7^ This solution to curbing the rapid escalation of obesity has been endorsed by the World Health Organization and others as a cost–effective policy solution, if retail prices increase sufficiently (10–20%) to reduce consumption.^8^^,^^9^ However, evidence on the effectiveness and fairness of these new sweetened beverage taxes remains limited.

In this paper, we investigated the hypothetical impact of the new tax for different income groups in the Philippines using extended cost–effectiveness analysis.^10^ This approach is important for a study in the Philippines, where economic inequalities persist and the consequences of public policy are not always clear. Some people, including industry representatives, have expressed concerns that taxes on direct consumption unfairly burden low-income consumers.^11^ The evidence on sweetened beverage taxation is insufficient to support this claim. This study therefore sought to fill a gap in the global pool of knowledge by examining the relative impact of the new tax^12^^–^^17^ on the health and financial well-being of households in the Philippines.

## Methods

### Overview

We used a method of extended cost–effectiveness analysis based on studies of increased tobacco taxes and other interventions.^10^^,^^18^^–^^20^ Extended cost–effectiveness analysis is a policy assessment method for estimating the impact on three major outcomes: (i) health benefits (i.e. the reduction in premature mortality); (ii) elimination of out-of-pocket expenditure by patients, reduced government expenditure on health and the financial risk protection associated with those reduced expenditures; and (iii) the consequences across socioeconomic groups (e.g. income quintiles). We applied the method to ascertain the consequences for different income groups of imposing a sweetened beverage tax in the Philippines.

### Estimation methods

#### Beverage tax, price elasticity and consumption

We converted the 6 Philippine pesos tax to a percentage (13%) based on a price of 45 Philippine pesos per litre of a regular cola drink, which was the mean price of sugar-sweetened soft drinks in the Philippines (available in the data repository).^21^ We then multiplied the percentage change in price with price elasticities to obtain the percentage change in quantity of cola consumed. In line with evidence from other low- and middle-income countries, we assumed that 100% of the price increase would be paid by consumers instead of by distributors or manufacturers (i.e. pass-through rate of 100%).^22^

We multiplied the cola consumption in each quintile by the own-price elasticity estimate for its respective quintile. Own-price elasticity is the change in quantity of a product purchased in response to a change in its price. As we did not have local price elasticity estimates, we used elasticity estimates of demand for sugar-sweetened beverages by income quintile from another middle-income country, Mexico^23^ (which are similar to estimates from Chile; available in the data repository).^21^ We used the pre-tax per capita daily consumption of sugar-sweetened beverages by age, sex and income quintiles from the Philippines Food and Nutrition Research Institute (available in the data repository).^21^

#### Disease incidence

We used a previously published mathematical model^16^^,^^24^ to estimate the effect of reduced consumption of sugar-sweetened beverages on disease incidence over the 20-year period 2018–2037. This model has been used to estimate the impact of sugar taxes on disease incidence and mortality in Australia,^15^ Canada^17^ and South Africa.^12^^–^^14^

Following previous methods,^12^^,^^16^ we first calculated the effect of reduced consumption on body mass index (BMI) by converting change in consumption into change in energy intake and translating this into impact on body weight. We assumed that sugar-sweetened beverages have an energy density of 1800 kilojoules (kJ) per litre.^13^ Reduction in consumption and energy intake was converted into change in body weight using an energy balance equation which stated that a daily energy change of 94 kJ was associated with a change of 1 kg in body weight for adults, assuming no change in physical activity.^25^ We used the change in body weight and average height of individuals in each age quintile category to obtain the change in BMI by age quintile (available in the data repository)^21^ BMI was modelled as lognormal distribution and a change in BMI will change the mean of the distribution.

Second, the changes in BMI were converted into incidence of type 2 diabetes mellitus, ischaemic heart disease and stroke using the potential impact fraction, defined as the “proportional reduction in the incidence of a certain disease, resulting from a specific change in distribution of a risk factor in the population at risk.”^26^ We obtained the baseline age-and sex-specific incidence, prevalence and case-fatality rates of the diseases from DisMod II software package (World Health Organization, Geneva, Switzerland). We used data on the relative risk of type 2 diabetes mellitus, ischaemic heart disease and stroke due to a unit increase in BMI from the Global Burden of Disease study^27^ and the change in BMI by age quintile category (from the first step) to derive the age-and sex-specific potential impact fraction estimates using the EpigearXL add-in for Microsoft Excel, version 14.0 (EpiGear International Pty Ltd, Brisbane, Australia). The baseline incidence rate was scaled by the potential impact fraction to obtain the incidence and mortality rate due to the intervention. The changes in the incidence and mortality rate after the intervention then formed the inputs into the cohort life-tables. Using Erstaz add-in to Microsoft Excel version 14.0 (EpiGear International Pty Ltd, Brisbane, Australia) the population was simulated to 100 years of lifetime or death to estimate the reduction in disease incidence, premature deaths and health-care costs over a 20-year period 2018–2037.

#### Health expenditure

We calculated the reduction in health expenditure associated with the reduction in disease incidence for both the households and the government. The Philippines Health Insurance Corporation (PhilHealth), a parastatal organization that operates the national health insurance scheme, provided the case rate for type 2 diabetes mellitus-related hospital admissions (International statistical classification of diseases and related health problems,^28^ ICD codes: E11.0, E11.1, E11.5 and E11.6), ischaemic heart disease (ICD code: I25) and stroke (ICD codes: I60, I61, I62, I63, I66, I67.4). The mean annual costs for these three admissions were 12 424 Philippine pesos, 10 468 Philippine pesos and 30 302 Philippine pesos, respectively. Due to lack of detailed age-, sex- and sector-specific costs in the PhilHealth database, we assumed that the mean cost applied uniformly across all age groups.

To estimate out-of-pocket payments by patients and government expenditure through subsidies to patients, we apportioned the health-care costs in each income quintile by the level of government financing and co-payment under insurance for each quintile. Health insurance is largely provided by PhilHealth and out-of-pocket payments are determined by age, employment and income. Out-of-pocket payments form a major proportion of health care financing in the Philippines, and attempts have been made to address this, especially for the lowest income quintiles. As a result, the bottom quintile only pays 20% of their health-care costs out-of-pocket, whereas the highest quintile pays up to 83% of the costs. Government share of financing health-care costs also varies by income quintiles and while government provides 67% of financing for the lowest income quintile, its share of financing drops to only 8% for the highest income quintile.^29^ Using this estimate of out-of-pocket payments and government financing as a proportion of health-care costs, we estimated the reduction in out-of-pocket payments and government expenditure for each averted case of type 2 diabetes mellitus, ischaemic heart disease and stroke.

#### Financial risk protection

For financial risk protection, we estimated catastrophic health expenditure (disease expenditure exceeding 10% of total yearly household expenditure) and the number of individuals avoiding disease-related catastrophic health expenditure after implementation of the tax.^30^

#### Additional tax revenues

We estimated the total change in tax revenue due the tax and calculated the proportion of this change borne by each income quintile. We used sugar-sweetened beverage consumption at baseline and the mean price (45 Philippine pesos) of a litre of sugar-sweetened beverages to calculate the post-policy tax revenue.

### Data sources

We obtained the total population by age and sex, and income quintile for 2013 from the Philippines Statistics Authority and the distribution by income quintile from the Philippines Demographic and Health Survey 2013. We obtained the BMI, the mean height of the population and sugar-sweetened beverages consumption by age, sex and income quintile from the Philippines National Nutrition Survey 2013. The baseline characteristics and the inputs are shown in Box 1.

Box 1 **Input parameters used in the extended cost–effectiveness analysis of the sweetened beverages tax in the Philippines**

**Size of population**

98.2 billion (Philippines Statistics Authority, 2013).

**Daily consumption of sugar-sweetened beverages, by income quintile**

Quintile 1 (poorest): 0.13 L; quintile 2: 0.18 L; quintile 3: 0.21 L; quintile 4: 0.26 L; quintile 5 (richest): 0.29 L (National Nutrition Survey, 2013).

**Average proportion of health-care costs as out-of-pocket payments, by income quintile**

Quintile 1: 20% (Philippine pesos 424/2093); quintile 2: 37% (Philippine pesos 932/2528); quintile 3: 52% (Philippine pesos 1741/3358); quintile 4: 71% (Philippine pesos 4211/5945); quintile 5: 83% (Philippine pesos 11640/14007; Philippines National Health Account, 2013 as cited in Racelis at al.).^29^

**Income per capita quintiles**

Quintile 1: Philippine pesos ≤ 23  523 (US$ 470); quintile 2: Philippine pesos  23 524–35 886 (US$ 470–718); quintile 3: Philippine pesos  35 887–53 943 (US$ 718–1079); quintile 4: Philippine pesos  53 944–91 136; (US$ 1079–1823); quintile 5: Philippine pesos > 91 136 (US$ 1823; Family Income and Expenditure Survey 2015).

**Gross domestic product (nominal price)**

15 806.4 billion Philippine pesos (Philippines Statistics Authority, 2015).

US$: United Sates dollars.

### Sensitivity analysis

We conducted three univariate sensitivity analyses. First, we reduced the pass-through effect from 100% to 50%. Second, we increased the pass-through effect to 150%. The pass-through effect could vary substantially across countries, across retailers within the country and across time. A study in the United States showed that retail prices of sugar-sweetened beverages in areas where a tax was implemented increased by 61% in the first month followed by 93% in the second month.^22^ Third, we used a uniform price elasticity measure across all income quintiles by applying a mean price elasticity of −1.166 across all income quintiles. We obtained this figure by calculating a simple average of elasticity values across the five income quintiles from Mexico (available in the data repository).^21^ This helped us to see the health effect due to differences in consumption and risk factors at baseline and on health-care costs due to differences in subsidy levels across the income quintiles.

## Results

We present the results by income quintile on the number of premature deaths due to type 2 diabetes mellitus, ischaemic heart disease and stroke; the reduction in out-of-pocket payments; the additional tax revenue generated; and the financial risk protection obtained. The estimates for health-care costs and tax revenues are in nominal terms, meaning that they do not account for price inflation. We also did not apply a discount rate to convert future costs into present value.

**Fig. 1:**
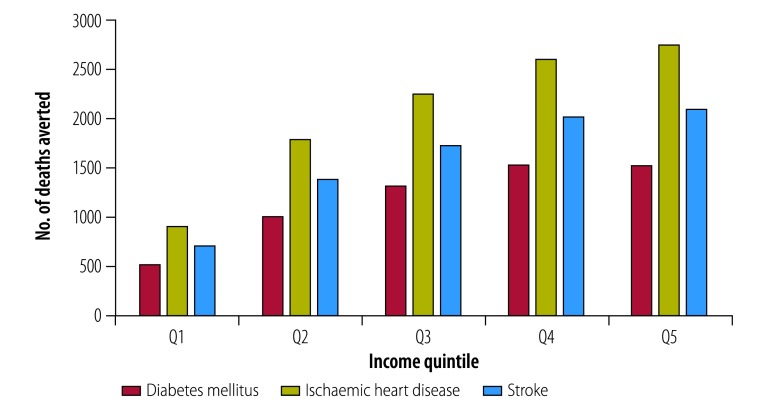
Projected potential deaths averted due to diabetes mellitus, ischaemic heart disease and stroke after implementation of the sweetened beverages tax in the Philippines, 2018–2037

Notes: Q1: lowest income quintile, Q5: highest income quintile. From January 2018 the tax on sweetened beverages was levied at 6 Philippine pesos per litre (United States dollars: 0.12).

Fig. 1 shows the number of premature deaths averted due to the new tax, projected over 20 years. We estimated that 5913 type 2 diabetes mellitus-related deaths, 10 339 ischaemic heart disease-related deaths and 7950 stroke-related deaths could be averted. The impact was more pronounced in the fourth and fifth income quintiles of the population, with around half of the overall deaths averted in these two quintiles. The smallest effect, with around 10% of overall deaths averted, was among the lowest quintile, who had relatively lower consumption of sugar-sweetened beverages at baseline.

**Fig. 2:**
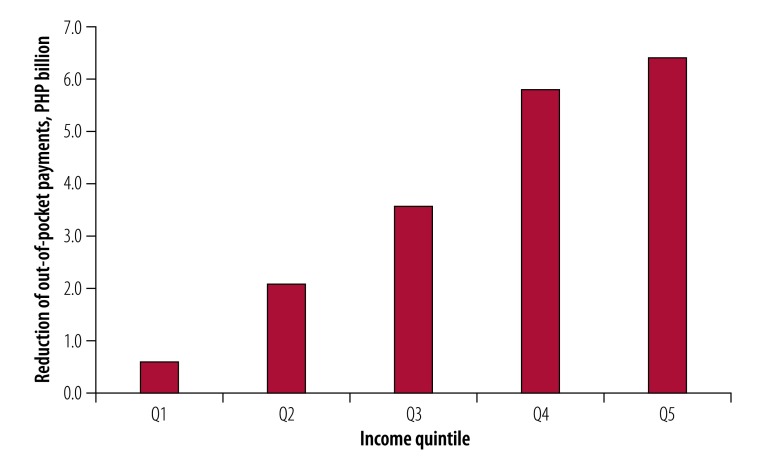
Projected reduction of out-of-pocket health-care payments by income quintile after implementation of the sweetened beverages tax in the Philippines, 2018–2037

We projected a reduction of 31.6 billion Philippine pesos (US$ 627 million) in health-care costs over the 20-year period (Table 1) and a total out-of-pocket cost savings of 18.6 billion Philippine pesos (US$ 369 million) over the same period (Table 2). Given the unequal distribution of out-of-pocket payments across quintiles, the highest income quintile could save the most (6.4 billion Philippine pesos; 35% of total out-of-pocket savings) while the lowest income quintile could save the least (0.6 billion Philippine pesos; 3% of total out-of-pocket savings; Fig. 2).

**Table 1:** Summary findings for the extended cost–effectiveness analysis of the sweetened beverages tax in the Philippines

Variable	Total	Quintile 1	Quintile 2	Quintile 3	Quintile 4	Quintile 5
No. of diabetes mellitus incident cases averted	299 540	28 917	55 289	66 045	76 960	72 329
No. of diabetes mellitus deaths averted over 20 years	5 913	522	1 006	1 321	1 532	1 532
No. of ischaemic heart disease incident cases averted	40 882	3 594	7 149	8 881	10 280	10 978
No. of ischaemic heart disease deaths averted over 20 years	10 339	908	1 794	2 259	2 616	2 762
No. of stroke incident cases averted	19 858	1 768	3 454	4 302	5 013	5 321
No. of stroke deaths averted over 20 years	7 950	705	1 387	1 732	2 022	2 104
Total health-care savings^a^ over 20 years, billion Philippine pesos	31.6	3.0	5.7	6.9	8.2	7.8
Total reduction in out-of-pocket payments over 20 years, billion Philippine pesos	18.6	0.6	2.1	3.6	5.8	6.4
Changes in annual tax revenues, billion Philippine pesos	41.0	5.6	7.0	8.0	9.9	10.5
No. of cases of catastrophic expenditure averted	13 890	8 269	1 953	2 184	1 484	0

^a^ Total health-care savings include savings on government costs and patients’ out-of-pocket payments.

Notes: The estimates for health-care costs and tax revenues do not account for discounting and are in nominal terms. From January 2018 the tax on sweetened beverages was levied at 6 Philippine pesos per litre (United States dollars: 0.12). We projected effects over the 20-year period 2018–2037

**Table 2:** Summary findings for the sensitivity analysis of the pass-through effect for the extended cost–effectiveness analysis of the sweetened beverages tax in the Philippines

Variable	Total	Quintile 1	Quintile 2	Quintile 3	Quintile 4	Quintile 5
**Pass-through effect reduced to 50%**						
No. of diabetes mellitus incident cases averted	164 162	15 729	30 294	36 305	42 153	39 681
No. of diabetes mellitus deaths averted over 20 years	3 091	251	514	702	814	810
No. of ischaemic heart disease incident cases averted	22 037	1 934	3 887	4 801	5 522	5 893
No. of ischaemic heart disease deaths averted over 20 years	5 574	488	976	1 221	1 405	1 484
No. of stroke incident cases averted	10 691	949	1 873	2 311	2 712	2 846
No. of stroke deaths averted over 20 years	4 280	378	752	930	1 094	1 126
Total health-care cost savings^a^ over 20 years, billion Philippine pesos	17.3	1.6	3.1	3.8	4.5	4.2
Total reduction in out-of-pocket payments over 20 years, billion Philippine pesos	10.2	0.3	1.2	2.0	3.2	3.5
Changes in annual tax revenues, billion Philippine pesos	44.7	6.1	7.8	8.8	10.7	11.3
No. of cases of catastrophic expenditure averted	7 483	4 490	1 048	1 124	821	0
**Pass-through effect increased to 150%**						
No. of diabetes mellitus incident cases averted	410 108	40 156	75 161	89 891	104 876	100 024
No. of diabetes mellitus deaths averted over 20 years	8 225	759	1 401	1 819	2 106	2 140
No. of ischaemic heart disease incident cases averted	57 185	5 030	9 912	12 389	14 371	15 483
No. of ischaemic heart disease deaths averted over 20 years	14 466	1 277	2 486	3 150	3 657	3 896
No. of stroke incident cases averted	27 819	2 499	4 802	5 990	7 043	7 485
No. of stroke deaths averted over 20 years	11 137	997	1 927	2 410	2 842	2 961
Total health-care cost savings^a^ over 20 years, billion Philippine pesos	43.3	4.2	7.8	9.4	11.2	10.7
Total reduction in out-of-pocket payments over 20 years, billion Philippine pesos	25.5	0.8	2.9	4.9	7.9	8.9
Changes in annual tax revenues, billion Philippine pesos	37.3	5.1	6.2	7.2	9.0	9.7
No. of cases of catastrophic expenditure averted	19 202	11 513	2 667	2 974	2 048	0

^a^ Total health-care savings include savings on government costs and patients’ out-of-pocket payments.

Notes: The estimates for health care costs and tax revenues do not account for discounting and are in nominal terms. From January 2018, the tax on sweetened beverages was levied at 6 Philippine pesos per litre (United States dollars: 0.12). We projected effects over the 20-year period 2018–2037 Pass-through rate determines how much of the increase in tax is passed to consumers as an increase in retail prices instead of being absorbed or paid by distributors or manufacturers. Due to rounding, percentages may not total 100%.

PHP: Philippine pesos.

Notes: Q1: lowest income quintile, Q5: highest income quintile. From January 2018 the tax on sweetened beverages was levied at 6 Philippine pesos per litre (United States dollars: 0.12).

**Fig. 3:**
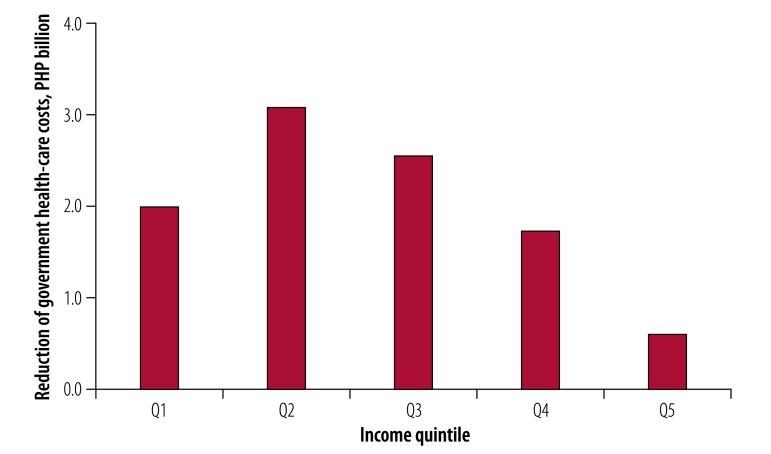
Projected reduction of government health-care costs by income quintile after implementation of the sweetened beverages tax in the Philippines, 2018–2037

Projected government contributions to health-care costs also differed across income quintiles. Due to progressive policy, government expenditure on health (government schemes and compulsory contributory health-care financing schemes) contributed to 67% (37 403 of 55 557 Philippine pesos) of health-care costs in the lowest quintile and 8% (16 117 of 190 521 Philippine pesos) for the highest quintile, with an overall contribution of 28% (130 028 of 465 241 Philippine pesos) across the quintiles (Table 2). This distribution of funding across quintiles is reflected in the distribution of savings across quintiles, as the tax could contribute to 10 billion Philippine pesos (US$ 198 million) in savings over 20 years and 57% (3.1 billion + 2.6 billion Philippine pesos) of these savings could be from quintiles 2 and 3 (Fig. 3).

PHP: Philippine pesos.

Notes: Q1: lowest income quintile, Q5: highest income quintile. From January 2018 the tax on sweetened beverages was levied at 6 Philippine pesos per litre (United States dollars: 0.12).

**Fig. 4:**
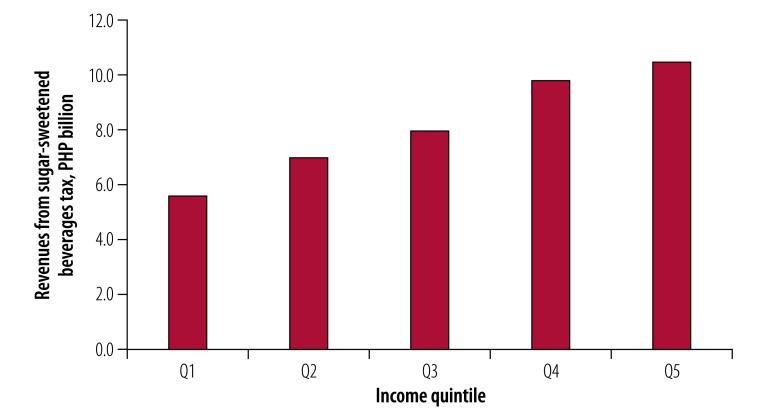
Projected annual revenues from the sweetened beverages tax by income quintile in the Philippines, per annum, 2018–2037

In addition, we estimated that there could be a net increase in annual tax revenues, with the government receiving an additional 41.0 billion Philippine pesos per annum (0.26% of the 2015 nominal gross domestic product of 15 806.4 billion Philippine pesos). Fig. 4 shows that the lowest income quintile could bear the smallest proportion (14%, 5.6 billion Philippine pesos) of this increase in tax burden while the highest income quintile could bear the largest share (26%, 10.5 billion Philippine pesos).

PHP: Philippine pesos.

Notes: Q1: lowest income quintile, Q5: highest income quintile. From January 2018 the tax on sweetened beverages was levied at 6 Philippine pesos per litre (United States dollars: 0.12).

To measure financial risk protection, we estimated that the tax could avert 13 890 cases of catastrophic health expenditure.

### Sensitivity analysis

When we reduced the pass-through effect to the lower bound of 50%, we observed changes in effects in both absolute terms and in the distribution across income quintiles. First, we estimated that the number of type 2 diabetes mellitus-related premature deaths averted over 20 years could be reduced to 3091 (a reduction of 47%), while ischaemic heart disease and stroke-related deaths decline to 5574 (46% reduction) and 4280 (46% reduction), respectively (Table 2). In contrast to the reduction in premature deaths, we projected an increase in tax revenues to 44.7 billion Philippine pesos. Lastly, we estimated that 7483 cases of catastrophic health expenditure would be averted (an 46% reduction).

Applying a price elasticity of −1.166 across all income quintiles we projected that the proportion of overall type 2 diabetes mellitus deaths averted for quintile 5 could increase to 30% (1811 of 6056 deaths) from 26% (1532 of 5913 deaths) in the baseline scenario (Table 3). A similar pattern was observed for stroke and ischaemic heart disease-related deaths, and the shift occurred from quintile 2 to quintile 5. The total tax revenue and distribution of its burden across income quintiles was roughly similar to the baseline scenario.

**Table 3:** Summary findings for the sensitivity analysis for elasticity for the extended cost–effectiveness analysis of the sweetened beverages tax in the Philippines, 2018–2037

Variable	Total	Quintile 1	Quintile 2	Quintile 3	Quintile 4	Quintile 5
**Mean elasticity of −1.166 applied across quintiles **						
No. of diabetes mellitus incident cases averted	305 269	29 946	46 495	62 377	81 316	85 135
No. of diabetes mellitus deaths averted over 20 years	6 056	546	835	1 244	1 620	1 811
No. of ischaemic heart disease incident cases averted	42 087	3717	6 018	8396	10 932	13 024
No. of ischaemic heart disease deaths averted over 20 years	10 646	940	1 510	2 135	2 782	3 279
No. of stroke incident cases averted	20 427	1833	2 911	4 037	5 352	6 294
No. of stroke deaths averted over 20 years	8 172	731	1 167	1 624	2 160	2 490
Total health-care cost savings over 20 years, billion Philippine pesos^a^	32.2	3.1	4.8	6.5	8.7	9.1
Total reduction in out-of-pocket payments over 20 years, billion Philippine pesos	19.5	0.6	1.8	3.4	6.1	7.6
Changes in annual tax revenues, billion Philippine pesos	40.9	5.6	7.3	8.1	9.7	10.2
No. of cases of catastrophic expenditure averted	13 826	8 556	1 632	2 011	1 627	0

^a^ Total health-care savings include savings on government costs and patients’ out-of-pocket payments.

Notes: The estimates for health care costs and tax revenues do not account for discounting and are in nominal terms. From January 2018 the tax on sweetened beverages was levied at 6 Philippine pesos per litre (United States dollars: 0.12). We projected effects over the 20-year period 2018–2037 Own price elasticity of demand of a good is the change in quantity demanded of the good in response to a change in its own price. We obtained the mean elasticity of −1.166 by calculating a simple average of elasticity values across the five income quintiles from Mexico (−1.12 in Q1, −1.41 in Q2, −1.24 in Q3, −1.09 in Q4, −0.97 in Q5 (available in the data repository).^21^

## Discussion

Our analysis showed that an excise tax of around 13% on sweetened beverages in the Philippines may generate population-level health gains. We demonstrated that the wealthiest quintiles will be most affected by the tax. This differs from a recent study in Mexico that demonstrated that the reductions in consumption were higher among the lower socioeconomic status group (10%) than among the high socioeconomic status group (6%)^31^^,^^32^ and the maximum reduction in BMI was obtained in the lowest levels of socioeconomic status.^33^ Similarly, in Australia, it was estimated that a 20% tax would lead to almost 50% of the gains within the lowest income quintiles.^15^ While the findings from our study differ from those studies, as to which segment of the population benefits, they all illustrate that improvements can be made in health-care promotion through taxation.

This analysis of the relative impact of such a tax illustrates the power of regulation of sugar consumption in the studied contexts. On the one hand, sugar plays a powerful role in fuelling the obesity burden and related health conditions. On the other hand, regulating sugar proves to be an effective tool for curbing consumption, and importantly this tax does not appear to function as a regressive imposition on the poor. In fact, the tax evaluated in this study reflects pro-poor health financing in the Philippines. As such, the tax burden would progressively increase, with the bottom two income quintiles bearing about 30% of the tax burden. This is especially important in low- and middle-income countries, where noncommunicable diseases are rising.^34^

Therefore, our research contributes timely evidence to suggest that sugar-sweetened beverage taxes are not universally regressive and can be compatible with health-system goals that include the progressive attainment of universal health coverage. Our findings suggest that distributional benefits of these taxes reflect not only a country’s underlying level of domestic consumption, but also the degree to which the health system has installed measures of financial protection for low-income households. Furthermore, sugar-sweetened beverage taxes are a way for countries to raise revenues, a hard-to-achieve policy priority of low- and middle-income countries’ health systems.

Taxing sugar-sweetened beverages is a political undertaking. Taxation policy development involves cooperation among an array of influential actors who have different interests. Many countries combatting the growing threat of noncommunicable diseases also benefit economically through domestic sugar production, consumption and international trade. These forces are historical, socially contingent and often path-dependent as they are tied to the political trajectories of decision-makers, including elected officials. Nevertheless, in this new area of research, we still do not know enough about how industry and the government can work together to strengthen the health and well-being of citizens.^35^ Of all the policy interventions to curb consumption of harmful products, from marketing restrictions to warning labels and manufacturing regulations, taxing sugar-sweetened beverages may prove to be the most useful at present. Some countries are experimenting with tax structures to incentivize reformulation of sugar-sweetened drinks^36^ and the possibility of taxation may even facilitate self-regulation by the beverage industry.^37^

Early evidence suggests that health advocates need to remain vigilant to ensure that sugar-sweetened beverage taxes endure. In the United States, a tax in Cook County (which includes the metropolitan area of Chicago) was repealed after two months.^38^ Beverage manufacturers undermined Berkeley, California’s sugar-sweetened beverage tax by passing a pre-emptive state-wide ban on other local sugar-sweetened beverage taxes.^39^ Borrowing tactics from tobacco and alcohol, the food and beverage industry in Mexico continues its efforts to counteract the Mexican sugar-sweetened beverage tax in several ways.^40^ All indications are that the sugar-sweetened beverage tax in the Philippines will face similar challenges. For example, an influential sugar-sweetened beverage manufacturer in the Philippines announced layoffs of employees only weeks after passage of the new tax.^41^ Similarly, an international producer of sugary powder mixes has threatened to relocate its manufacturing business elsewhere in response to the Philippines tax.^42^ These examples underscore the importance of using sound evidence to provide arguments in support of sugar-sweetened beverage taxation and its role in reducing noncommunicable diseases.

Our study has several limitations. First, we did not have direct estimates of the price elasticity of sugar-sweetened beverage consumption by income quintile for the Philippines. Instead, we used estimates from Mexico because the countries are similar in important ways, such as their tropical geographical locations that underpins food-chains, shared colonial legacy that affects culture, diet and language, and common trade partners that influence dietary patterns. Second, we did not have cross-price elasticity estimates for substitutes such as milk and fruit juices. We do not expect that individuals would switch to non-caloric drinks such as water but would likely switch to other untaxed drinks. Third, we did not include the 12 Philippine pesos per litre tax on sugar-sweetened beverages made with high-fructose corn syrup because we did not have access to data on the composition of all sugar-sweetened beverages available in the Philippines. The two-tiered tax structure may encourage product reformulation, which our model is unable to accommodate. Fourth, we did not have data on variations in health-care use by income quintile and disease condition and we assumed 100% utilization of health-care facilities by those with any disease condition. Fifth, due to lack of data on costs in primary-care setting we used health-care costs associated with inpatient settings, whereas for several noncommunicable diseases, the care could be, and often is, managed in primary-care settings. Lastly, we did not consider non-medical costs such as loss of productivity, transportation costs and caregiver costs.

This study contributes to the growing base of evidence^43^ to suggest that sugar-sweetened beverage taxation can be a cost–effective means of addressing the growing threat of noncommunicable disease in low- and middle-income countries. However, there remains a need for empirical research from the Philippines and elsewhere to understand the impact of new sweetened beverage taxes on different income groups after implementation. How this evidence is used to inform debate in the Philippines and in other countries is political and difficult to predict. Nevertheless, we argue that methods such as extended cost–effectiveness analysis can help inform the discourse on health-system strengthening and its role in poverty alleviation globally.
